# Structural diversity of p63 and p73 isoforms

**DOI:** 10.1038/s41418-022-00975-4

**Published:** 2022-03-21

**Authors:** Christian Osterburg, Volker Dötsch

**Affiliations:** Institute of Biophysical Chemistry and Center for Biomolecular Magnetic Resonance, Goethe University, Frankfurt am Main, Germany

**Keywords:** X-ray crystallography, Solution-state NMR

## Abstract

**Abstract:**

The p53 protein family is the most studied protein family of all. Sequence analysis and structure determination have revealed a high similarity of crucial domains between p53, p63 and p73. Functional studies, however, have shown a wide variety of different tasks in tumor suppression, quality control and development. Here we review the structure and organization of the individual domains of p63 and p73, the interaction of these domains in the context of full-length proteins and discuss the evolutionary origin of this protein family.

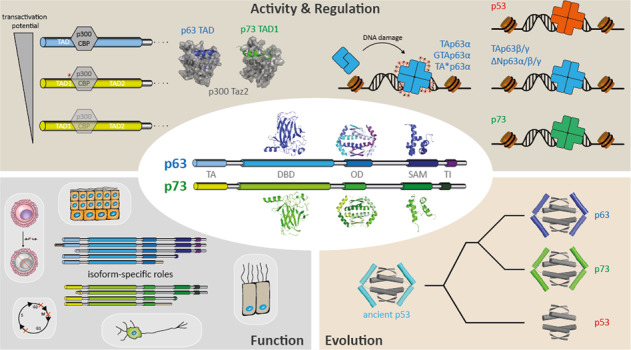

**Facts:**

- Distinct physiological roles/functions are performed by specific isoforms.
- The non-divided transactivation domain of p63 has a constitutively high activity while the transactivation domains of p53/p73 are divided into two subdomains that are regulated by phosphorylation.
- Mdm2 binds to all three family members but ubiquitinates only p53.
- TAp63α forms an autoinhibited dimeric state while all other vertebrate p53 family isoforms are constitutively tetrameric.
- The oligomerization domain of p63 and p73 contain an additional helix that is necessary for stabilizing the tetrameric states. During evolution this helix got lost independently in different phylogenetic branches, while the DNA binding domain became destabilized and the transactivation domain split into two subdomains.

**Open questions:**

- Is the autoinhibitory mechanism of mammalian TAp63α conserved in p53 proteins of invertebrates that have the same function of genomic quality control in germ cells?
- What is the physiological function of the p63/p73 SAM domains?
- Do the short isoforms of p63 and p73 have physiological functions?
- What are the roles of the N-terminal elongated TAp63 isoforms, TA* and GTA?

## Introduction

**Fig. 1:**
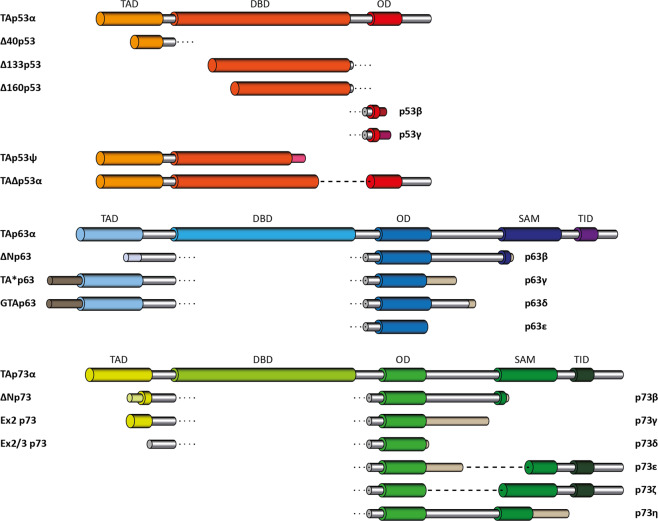
Domain organizations and isoforms in the p53 protein family. For p53, p63 and p73 all so far known isoforms are shown. TAD transactivation domain, DBD DNA binding domain, OD oligomerization domain, SAM Sterile-alpha motif domain, TID transactivation inhibitory domain. Except for TAp53α and Δ40p53α, all p53 isoforms contain incomplete segments of either the DBD or the OD which leads to unfolding of the corresponding domains. For p73 isoforms with a truncated SAM domain have been found as well.

The world of the tumor suppressor p53 was firmly anchored within the field of cancer biology but in the late 1990s this exclusive connection was questioned by the discovery of two proteins, p73 [1] and p63 [2–5], with high sequence similarity. All three proteins share a very similar DNA binding domain (DBD) in which virtually all amino acids that are known from p53 to contact DNA are conserved [5]. The initial impulse of assigning both proteins as additional tumor suppressor proteins was, however, questioned by the analysis of the p73 [6]- and p63 [7, 8]-knock out mice both of which show strong developmental abnormalities. Genetic deletion of p73 in mice causes severe neurodevelopmental defects including hippocampal dysgenesis, hydrocephalus and pheromone sensing impairments as well as chronic infections, inflammation and infertility [6, 9–11]. Detailed analysis of these mice have uncovered the role of p73 in development and maintenance of neurons, its role of an essential transcription factor for multiciliogenesis [12, 13], for sperm cell maturation [10, 11] and regulation of metabolism [14, 15]. The p63-knock out mouse suffers from even more severe developmental defects that include limb truncations and lack of a multi-layered skin and other epithelial structures [7, 8]. Analysis of wild type and knock out mice showed that p63 is highly expressed in the basal layer of stratified epithelial tissues, which is necessary to build up these multi-layered structures [16, 17]. Further mouse studies identified additional functions for p63 in metabolism [18–20], muscle development [21–23] and in particular in genetic quality control of oocytes [24]. These surprisingly diverse spectra of both proteins’ functions became clearer with the assignment of distinct isoforms to specific cellular tasks. Both p73 [1, 25–28] and p63 [5] are expressed in a whole array of different isoforms that are in both cases created by a combination of different N-terminal promoters and C-terminal splice variants (Fig. 1). For both genes the expression in different human and mouse tissues [29, 30] was investigated at the mRNA level that showed transcripts of several isoforms in most tissues with a greater diversity for isoforms of p73. However, the physiological importance of these different transcripts and if they are translated into protein is not known. Only for two p63 isoforms a physiological role is well defined: The isoform that is found in the epithelial tissue is ΔNp63α [7, 8, 16], which lacks the N-terminal transactivation (TA) domain, while TAp63α is the isoform expressed in oocytes [31, 32]. Both isoforms contain three folded domains: the DBD, the oligomerization domain (OD) and the sterile-alpha-motif (SAM) domain. TAp63γ, that is found in muscle tissue lacks the C-terminal SAM domain and contains a unique C-terminus [5]. All p63 isoforms differ only in the presence or absence of sequences that are intrinsically disordered in isolation. This is in stark contrast to some p53 isoforms that contain only parts of either the DBD or of the OD [33–36] (Fig. 1). As these residual DBD and OD sequences can no longer fold into a defined three-dimensional structure, these p53 isoforms contain large unstructured stretches, exposing in case of the unfolded DBD aggregation prone sequences [37–40] that are in wild type p53 hidden within the folded structure.

For p73 the assignment of physiological functions to well defined isoforms is less clear. In the brain both TAp73 and ΔNp73 isoforms are often expressed in the same neuronal cell types (except in the marginal zone which only expresses ΔNp73 [6]). In general, TAp73 seems to promote terminal neuronal differentiation by transcriptionally regulating neurotrophin receptor p75 [41] and microRNA miR34a [42]. ΔNp73 on the other hand plays a pro-survival role in discrete neuron types such as neurons in the preoptic area, as well as vomeronasal, GnRH-positive and Cajal–Retzius (CR) neurons [43]. Regulation of the transcriptional program important for multiciliogenesis [12, 13] as well as for contacts between developing sperm cells and nurturing Sertoli cells [10, 11] is governed again by TAp73 and TAp73 also plays a role in granulosa cells where it orchestrates a transcriptional program important for cell adhesion and cellular contacts to oocytes [44]. Finally, ΔNp73 is expressed in the basal layer of epithelial tissues as well [30, 45, 46], albeit at lower levels than p63. With respect to the C-termini, the most highly expressed isoform seems to be in most cases the α-C-terminus. In contrast to p63, however, the β-C-terminus is also often expressed to a significant level (for example up to 20% in skin) [45].

Recent progress in our understanding of the specific conformations adopted by different isoforms have advanced our insight into the regulation of their distinct functions. Here we review the structures of the individual domains, their structural and functional interplay with the sequences surrounding these domains and discuss a potential evolutionary path. As the conformation—function relationship is better understood for p63 than for p73, a focus of this review will be on p63. As the structure and function of p53 domains have been reviewed many times [47–50], we include short comparisons and discuss differences to the corresponding p53 domains but do not review p53 related issues in detail.

## Domains

### Transactivation domain

**Fig. 2:**
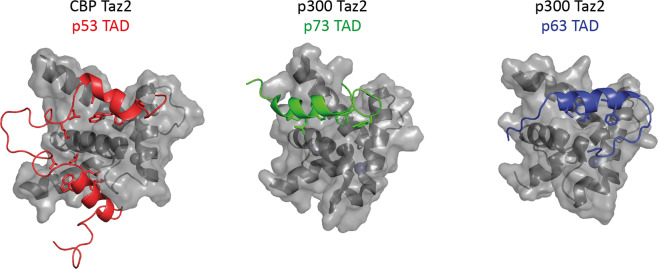
Structures of the TA domains of all family members bound to the Taz2 domains of either CBP (p53) or p300 (p73 and p63). The Taz2 domains are shown as gray surfaces with the underlying α-helices in dark gray. The TA domains are presented in red (p53), green (p73) and blue (p63). In case of p53, both the TAD1 and TAD2 bind simultaneously (PDB code: 5HPD). The p73 TAD1 binds in a different location; the position of the TAD2 could so far not unambiguously be identified (PDB code: 6FGS). p63 contains a single TA domain that is longer than the individual TA subdomains of p53 and p73 and binds to the same site as the p53 TAD2, albeit with a slight reorientation (PDB code: 6FGN).

Of all domains of p63 the N-terminal TA domain shows the lowest sequence identity to p53 but also to p73. Structurally the TA domains of all three family members are intrinsically unfolded when isolated [51, 52], but can show residual α-helicity in local segments [53, 54] which increases to form α-helices upon binding to interaction partners [55–66]. While usually p63 and p73 are closer related to each other than either to p53, the structural organization of the TA domain is the exception. The TA domains of p53 and p73 are divided into two subdomains (residues forming α-helices 16–25 and 47–55 in case of p53 and residues 15–20 and 61–65 in case of p73) [60, 67, 68]. Both subdomains of p53 and p73 bind simultaneously to different sites on the Taz1 and Taz2 (transcriptional adapter zinc-binding) domains of the transcriptional coactivator proteins CBP [68] (cyclic-AMP response element binding protein (CREB) binding protein) and p300 [61, 67] (Fig. 2A). The second subdomain of p53 (TAD2) interacts more strongly with the Taz1/Taz2 domains [68, 69] than the first subdomain (TAD1) but both synergize in the context of the full-length TA domain to enhance the binding affinity [67, 69] and drive different transcriptional programs [70–73]. In contrast to p53, in p73 the first subdomain is dominant for interaction with Taz1/Taz2 domains [60] and binds to a different site on the p300 Taz2 domain (Fig. 2B) [61]. In addition to the α-helical section (residues 15–20) two aromatic residues (Y28 and F29) located C-terminally to the α-helix contribute significantly to the interaction with the Taz2 domain [61]. The binding site of the second subdomain could not be determined unambiguously so far but likely involves the weaker binding site occupied by TAD1 in the p53 TA domain–Taz2 complex [61].

In p63 residues 8–25 form a single long helix that comprises the entire TA domain and that combines elements of both p53 subdomains [61]. No further sequences are required for high affinity binding to the Taz2 domain of p300 (Fig. 2C). Structurally, TAD2 of p53 and the TA domain of p63 occupy the same binding site on the Taz2 domains of CBP and p300, albeit at an angle of 26^o^ relative to each other. Since the TA domain of p63 is longer by one helical turn it forms additional hydrophobic contacts with the Taz2 domain.

Of all three family members the affinity of the TA domain of p73 to the Taz2 domain is the lowest, but can be enhanced by phosphorylation of T14 [60]. Similar to p73, phosphorylation of the p53 TA domain has been reported with multiple phosphorylation events enhancing the affinity to CBP in an approximately linear manner. Based on this observation a rheostat model has been proposed that allows p53 to respond gradually and not switch like to increasing levels of cellular stress [74]. In the case of p63, the unmodified TA domain already shows high affinity to the p300 Taz2 domain. The difference in affinity between the p73 and p63 TA domains is mirrored by the low transcriptional activity of TAp73β as compared to the high activity of TAp63γ in cellular transcriptional activity assays on the p21 promoter. Replacing the corresponding amino acids in the TA domain of p73 with residues 8–15 of the p63 TA domain confers the high transactivation potential and high affinity for the Taz2 domain of TAp63 onto TAp73 [61]. All these results predict that the transcriptional activity of the bipartite TA domains of p53 and p73 are at least partially regulated by phosphorylation while phosphorylation does not seem to be required for p63. Instead, the transcriptional activity of the longest TAp63α isoform is regulated via the oligomeric state with the sequence that is equivalent to TAD2 of p53 and p73 playing a crucial role (see below) [61].

In addition to interaction with domains of p300 and CBP the TA domains of all three family members also bind to the N-terminal domain of the E3 ligase Mdm2 (Mouse double minute 2 homolog). This interaction is very well characterized for p53 where it leads to ubiquitination via Mdm2’s RING finger domain and proteasomal degradation [75]. For this interaction the TAD1 subdomain is responsible [56]. Both subdomains can also bind simultaneously to TAZ1/TAZ2 (via TAD2) and to MDM2 (via TAD1) to form a ternary complex [69]. In contrast to the clear picture of the role of Mdm2 for the regulation of the activity of p53, the functional importance for p63 remains less clear [76–78]. Transcriptional repression [79] as well as transcriptional enhancement [76] or having no effect [77, 80] have been reported. For p73 binding of the TAD1 to Mdm2 leads to inhibition of its transcriptional activity [81–83] and probably contributes to the regulation of p73’s tumor suppressor activity [84–86]. One noticeable difference between p63 and p73 on the one hand and p53 on the other hand is that interaction with Mdm2 does not lead to ubiquitination and proteasomal degradation. Any potential regulatory effect is therefore limited to binding of Mdm2 to the TA domains and blocking interaction with transcriptional co-activators CBP and p300. Since p73 interacts with Mdm2 via its TAD1 subdomain and p63 contains only a single undivided TA domain, blocking these sites that are also crucial for interaction with CPB or p300 effectively inhibits transcriptional activity. In p53 the situation is different with TAD1 interacting with Mdm2 but not TAD2 which has a higher affinity for CBP/p300. Structures of the TAD1 of p73 and the TA domain of p63 with the N-terminal domain of Mdm2 have been solved [64] showing in both cases a similar binding mode compared to TAD1 of p53, involving the conserved FxxΦWxxL motif (Φ: Leu or Ile, x: any amino acid). However, p63 forms only a short one turn helix, while p73 and p53 fold into longer two turn helices which also results in more than a tenfold lower affinity of p63 to Mdm2 [64, 87, 88] and its hetero-oligomerization partner Mdmx [89] as compared to the other two family members.

### DNA binding domain

p53, p73 and p63 share the highest sequence identity in their DBD reaching 65%. Despite this high sequence conservation transcriptomic analysis has revealed differences in the genes regulated by the three proteins [90–92]. Furthermore, DNA binding studies have demonstrated differences in the sequence preference of p53 and p63 [93–95]. In two studies a guanosine base is allowed in position 5 of the p53 consensus sequence (RRRCWWGYYY; R = A or G; W = A or T; Y = C or T). While p53 preferred in these studies binding sites with a central CATG sequence, p63 binds strongly to both CATG- and CGTG-containing sequences with the highest preference for sequences containing the half-site 5′-RRRCGTGYYY-3′ [94, 95]. Another study based on a SELEX analysis found preferential binding to the core CATG sequence albeit with a preference for A/T-rich segments in the flanking regions (5′-(T/A)(A/T)AC(A/T)TGTTT-3′) [93].

**Fig. 3:**
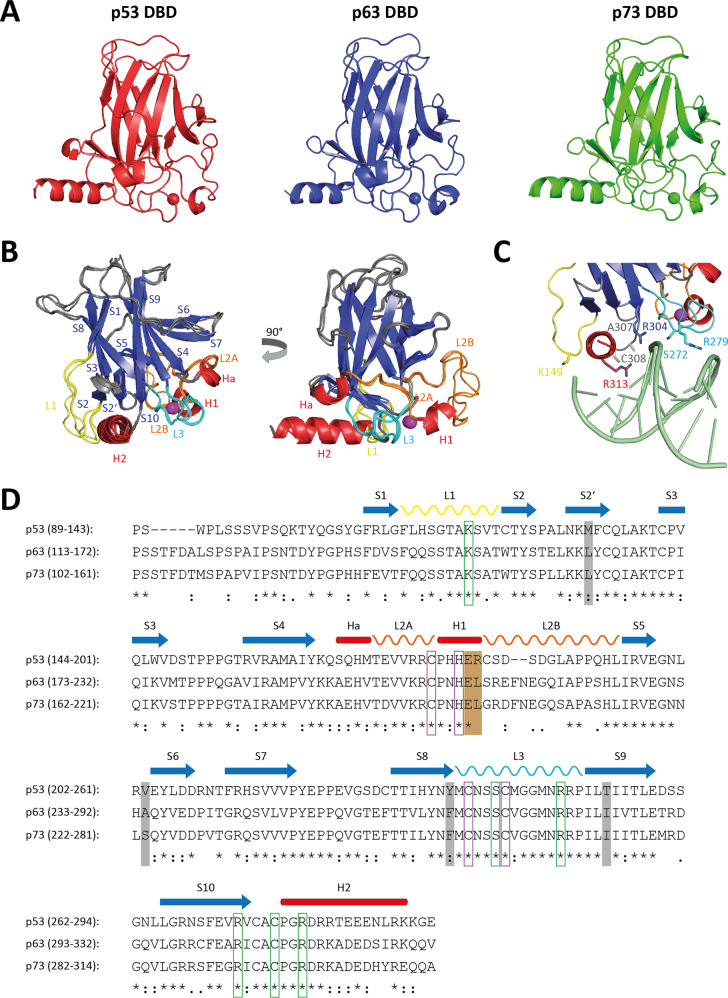
Structure, organization, and sequence similarity of the p53 family DBDs. **A** p53 (red, PDB code: 2AC0), p63 (blue, PDB code: 3QYM) and p73 (green, PDB code: 3DV0) DBDs consist of an immunoglobulin-like β-sandwich of two β-sheets as the domain scaffold and exhibit high structural homology. **B** Two different orientations of the superimposed p53 family DBDs with explicit labeling of the loop-sheet-helix DNA recognition element created by the L1 loop (yellow), the S2-S2’-S10 sheet (blue) and the H2 helix (red). The loop L3 that provides additional contacts is labeled in cyan. **C** Close-up of the interaction of the p63 DBD with the DNA. Critical amino acids are indicated. **D** The DBDs show high sequence identity and conservation of secondary structural elements and residues involved in scaffold assembly and DNA binding. They are composed of ten β-strands (S1–S10), three helices (Ha, H1 and H2) and four relevant loops (L1, L2A, L2B and L3). Residues directly contacting DNA and coordinating the structural important zinc ion are framed in green and purple, respectively. Amino acids responsible for thermodynamic stability differences are highlighted in gray. The two charged residues, which are forming a salt bridge in the intra-dimer interface of p53 and that are crucial for its DNA binding cooperativity, are marked in brown.

The structure of the p63 DBD has been solved in complex with DNA with both a 10-bp DNA half-site response element (5′AAACATGTTT3′) as well as with a 22-bp DNA full response element containing a 2-bp spacer between both half-sites (5′AAACATGTTTTAAAACATGTTT3′) [96]. Both DNA sequences contain the CATG core motif, flanked by A/T-rich sequences. The structures of the p53 and p63 DBD are very similar (root mean square deviation of 0.9 Å in α-carbon atom positions, Fig. 3A) and contact the DNA in a very similar manner, although different DNA sequences were used in the different studies of p53-DNA complexes [96–101]. Both domains contain a loop-sheet-helix motif that consists of the L1 loop, a three-stranded β-sheet (S2, S2′, S10) and the H2 α-helix (Fig. 3B). The residues that contact the DNA are conserved between both proteins with R311 (R280 in p53) providing essential contacts to the G7 base in the major groove and A307 (A276 in p53) and Cys308 (C277 in p53) interacting with the methyl group of T8. Further contacts are provided by Arg304 (R273 in p53) interacting with the backbone phosphate of T6 and the A307 (A276 in p53) amide group binding to the phosphate backbone of G7 (Fig. 3C, D). In contrast to the p53-DNA structures, the L1 loop is more disordered and the contact of K149 (K120 in p53) is missing. K120 of p53 is not one of the mutational hotspots and this residue as well as the L1 loop can be bound by iASPP which modulates the sequence specificity of DNA binding and shifts the p53 based transcriptional program, affecting genes involved in life/death decisions [102]. Outside of the loop-sheet-helix motif, residues in the L3 loop provide further DNA contacts. Ser272 (S241 in p53) interacts with the phosphate backbone of G7. Finally, the side chain of R279 potentially interacts with the DNA phosphate backbone of T20 or T21, but the distance is too far for a standard salt bridge. The corresponding residue in p53, R248, is the most frequently mutated residue found in cancer cells [103] and makes contacts in the minor groove of the A/T-rich region flanking the core CATG sequence which results in a narrowing of the minor groove [97].

In principle, the OD ensures that all family members interact with the DNA as tetramers, thus increasing binding affinity (exceptions are TAp63α and Cep1, see below). In addition, the individual DBDs can interact with each other using different interfaces. Cooperative binding of two DBDs to one half site is known from p53 where R181 and E180 in the H1 α-helix form two salt bridges between two monomers [104] to which P177 and H178 on the H1 α-helix as well as M243 in the L3 loop further contribute hydrophobic contacts [100, 101]. In p63 R181 is replaced with a leucine residue and accordingly the isolated p63 DBD does not show cooperative binding to DNA but requires either its own OD or an artificial oligomerization system (e.g., fusion to GST) [104, 105]. This results in a ~ three orders of magnitude lower binding affinity of the p63 DBD to DNA compared to the p53 DBD [96, 106]. Nevertheless, close contacts between two p63 DBDs bound to a half site have been identified in the crystal structures involving Asn207 that forms inter-monomer hydrogen bonds as well as Leu210 (both located in helix 1) and Val274 (located in loop L3) that are part of a small inter-monomer hydrophobic patch [96].

Crystal structures of the p53 DBD bound to various DNA oligomers have shown that protein-protein contacts also exist between two dimers, thus forming an additional tetramerization interface [98, 99, 101, 107], that however differs between individual complexes depending on the spacer length between the two half sites [100]. For p63 additional protein-protein contacts between dimers have been identified as well. Such contacts are visible, however, only in the crystal structure with the 10 bp DNA and are not observed in the structure of the 22 bp DNA with a 2 bp spacer between the two half sites [96]. Consequently, the affinity of the p63 DBD to this 22 bp DNA is even lower than to DNA without the spacer showing that dimer-dimer contacts contribute to DNA binding [108]. In contrast to the interface between two monomers within a dimer, the interface between dimers varies and several different interfaces have been observed in the crystal structure with the 10 bp DNA, which are also affected by crystal contacts [96]. Further crystal structures of the p63 DBD in complex with different DNA oligomers containing sequence variations in the spacer between the two half sites have demonstrated that the sequence of this spacer is important for the overall geometry of the protein-DNA complex [108]. Replacing the AT sequence with a GC spacer abolishes the superhelical DNA trajectory. These investigations underscore the importance of the DNA sequence and suggest that p63 may bind to superhelical DNA packed in nucleosomes. This ability to bind to DNA bound by nucleosomes was also reported for p53 that can bind to the distal p21 response element of mononucleosomal DNA if the binding site is not close to the diad center of the nucleosome [109]. As the ΔNp63α isoform plays an important role as an organizer of the chromosomal landscape in epithelial cells (see below), this ability might be important for its function.

The structure of the isolated DBD has been solved by liquid state NMR spectroscopy [110] as well showing some slight differences in the L1 loop, the L2 loop and the orientation of the H2 α-helix. These investigations have also revealed that the thermostability of the p63 DBD is with a melting temperature of ~60 °C significantly higher than the melting temperature of the p53 DBD (~43 °C) [105, 110, 111] and that of the p73 DBD (~50 °C) [111, 112]. Analysis of the hydrophobic cores of both p63 and p53 have shown that the core of p53 is less well packed with some cavities and unmatched polar residues [110]. Interestingly, these sites have been mutated in stability-optimized p53 variants [113, 114]. Since the p63 DBD is evolutionary older (see below) this result predicts that the low thermostability of p53 evolved later during evolution, potentially to support the fast degradation of cellular p53 which is under normal conditions kept at low levels [75].

Crystal structures were also published for the isolated p73 DBD [115], its complex with the pro-apoptotic protein ASPP2 (Apoptosis-Stimulating of p53 Protein 2) [115] and of the DBD in complex with DNA oligomers containing different sequences and spacer lengths between the two half sites [116, 117]. Recognition of the DNA sequence is very similar to the pattern observed for p63 and p53 with Lys138, Cys297 and Arg300 providing key contacts (Lys120, Cys277, and Arg280 in p53) (Fig. 3D). Interestingly, this study also found that the spacer length and sequence has a more pronounced effect on the transcriptional activity of p73 than on the transcriptional activity of p53, probably due to differences in contacts between the DBDs across the tetramerization interface. While the amino acids that directly contact the DNA are highly conserved within the p53 protein family, the sequences that provide protein-protein contacts important for forming dimers or tetramers when bound to DNA are less than 50% conserved between p73 and p53 [116]. Like in p63, the p53 salt bridges in helix H1 are missing and Met243 is replaced with Val263 in p73 which results in an overall smaller dimeric interface. The spacer length between both half sites influences in particular the interface between two dimers but also the dimeric interface between two monomers. The crystal structures of the p73 DBD bound to half sites separated by spacers with different lengths have revealed that—in contrast to p63—up to a 2 bp spacer a tetrameric interface exists. The increasing distance and different angle between the two dimers causes changes in the tetramerization interface and distortions of the DNA by partial unwinding within the spacer base pairs. How these structural changes translate into the reduced transcriptional activity of p73 (relative to p53) when bound to DNA with a spacer between the two half sites is, however, not understood but may contribute to the differences seen in transcriptional programs between the p53 family members [90, 118].

### Oligomerization domain

**Fig. 4:**
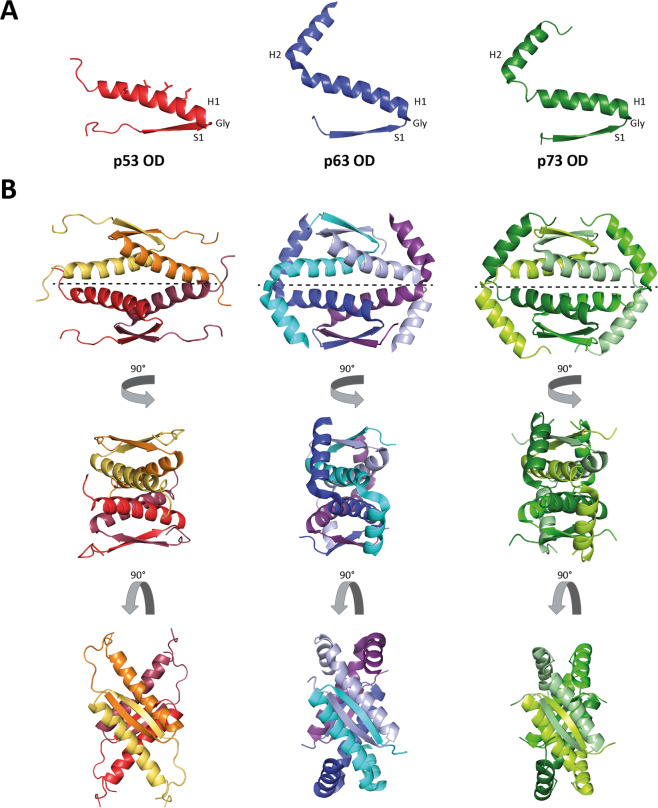
Structures of the oligomerization domains of all p53 family members. **A** The p53 oligomerization domain (OD) contains a β-strand S1 followed by an α-helix H1 (red). The p63 and p73 ODs are elongated by an addition helix H2 (blue and green). For p53 the residues constituting the nuclear export signal (NES) are depicted as sticks. In all three structures S1 is separated from H1 by a structurally important Gly residue. **B** Four monomers assemble into tetramers as a dimer of dimers with a D2 symmetry. One dimer is built by the formation of an antiparallel intermolecular β-sheet and an antiparallel two helix bundle. The hydrophobic surface presented by the helices engages in tetramerization leading to a four-helix bundle, thereby burying the NES. The second helix H2 of p63 and p73 stabilizes the tetramer further by reaching across the tetramerization interface and clutching the respective opposite dimer. The tetramerization interfaces are depicted by dashed lines (PDB code 1SAF, 4A9Z and 2KBY).

The ODs of all three members of the p53 family are dimers of dimers [119, 120]. The basic module consists of an antiparallel β-sheet formed by two monomers that is stabilized by two α-helices, one from each monomer, that also arrange in an antiparallel orientation (Fig. 4A). The link between the β-strand and the C-terminally following α-helix is tight with only a single glycine residue between both structural elements [119, 120]. Two of these modules form the final tetramer via the α-helical interface (Fig. 4B). Initially, structure determination of the p73 OD showed that this motif is extended by a second α-helix at the C-terminus [121, 122] that was later shown to exist in p63 as well [123]. This additional C-terminal α-helix reaches across the interface between both dimers and further stabilizes the tetrameric state (Fig. 4B). While this helix can be cleaved without unfolding the OD, its removal destabilizes the tetramer and shifts the equilibrium toward dimers [121, 122]. Surprisingly, further interaction studies have revealed that a mixture of p63 and p73 forms mixed tetramers with a p63_2_:p73_2_ tetramer being the thermodynamically most stable state (even more stable than both homo-tetramers) [121, 122]. The reason for this increased stability of the hetero-tetramer was identified from structure determination which demonstrated that the hydrophobic residues at the C-terminus of the second α-helix of p73 can interact with a hydrophobic patch at the β-strand of p63 [46]. Since p63 and p73 both are expressed in the basal layer of some epithelial tissues, specific functions for this hetero-tetramer might exist [30, 46]. The structural difference between the p53 OD on the one hand and the p63 and p73 ODs on the other hand also prevents stable interaction between p53 and the other family members [121, 122].

### Sterile-alpha-motif domain

**Fig. 5:**
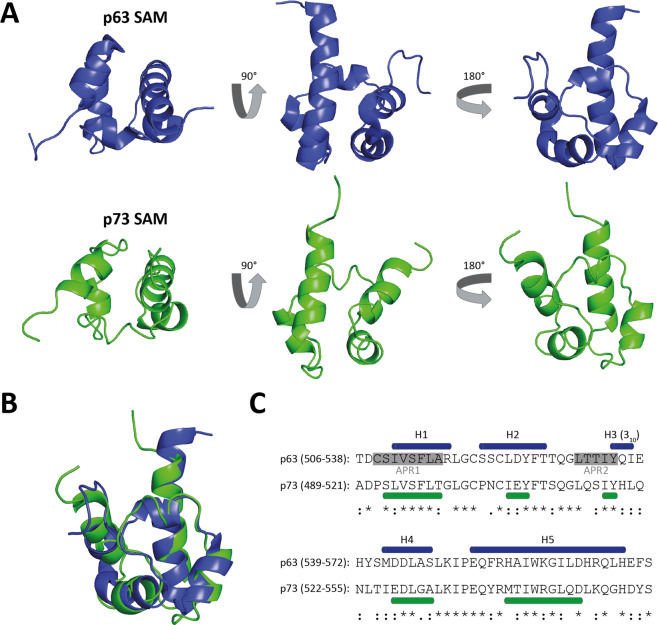
Structures of the p63 and p73 SAM domains. **A** The p63 SAM domain (blue; PDB code: 2Y9T) and the p73 SAM domain (green; PDB code: 1COK) are shown in different orientations. The domains each consist of four α-helices and one 3_10_-helix. **B** Superposition of the p63 and p73 SAM domains showing the high structural similarity. **C** Comparison of the p63 and p73 sequences of the SAM domains with indicated secondary structure elements. The two stretches in the p63 SAM domain with a high aggregation propensity are marked with gray. These sequences cause aggregation initiated by mutations in the SAM domain in patients suffering from ankyloblepharon-ectodermal defects-cleft lip/palate (AEC) syndrome.

The SAM domain which forms a compact structure consisting of five helices (four regular α-helices, one 3_10_-helix; Fig. 5A, B) exists only in p63 and p73 but is missing in p53 [124–126]. In general, SAM domains are found in a large variety of different proteins ranging from cell surface receptors (e.g., ephrin receptors), tankyrases to kinases and transcriptional repressors [127–130]. They have been implicated in homo- and hetero-oligomerization [131, 132]. The SAM domains of p63 and p73, however, show no tendency to homo-oligomerize [124, 126, 133]. The specific function of the p63 and p73 SAM domains still remains obscure. They have been proposed to bind to lipids [134], in particular ganglioside GM1 [135], as well as Mdm2 [136], the cyclin-dependent kinase binding protein Cables1 [137], the APOBEC1-binding protein ABBP1 [138] and the Rho GTPase activating protein DLC2 [139] but so far no consistent model of the function of these SAM domains has emerged.

Interestingly, mutations in the SAM domain of p63 have been linked to a developmental syndrome in human patients, the ankyloblepharon-ectodermal defects-cleft lip/palate (AEC) syndrome [140, 141]. Patients suffer from ankyloblepharon, congenital erythroderma, skin fragility, atrophy, palmoplantar hyperkeratosis, and extensive skin erosions [142]. Structure determination of the disease causing SAM domain mutant L514F has not revealed significant structural changes that could explain the disease phenotype [143]. Investigation of the thermodynamic stability of this as well as other SAM domain mutants has shown that all are thermodynamically less stable [143, 144]. However, some of them, including the L514F mutant, still have melting temperatures that are much higher than for example the melting temperature of the p53 DBD and within the same range as the p63 DBD [110]. The disease mechanism only became evident through refolding experiments of thermally unfolded SAM domains. While the wild type SAM domain refolds readily, the L514F mutant SAM domain does not refold but instead aggregates [143]. Analysis of the amino acid sequence of the SAM domain revealed the existence of two aggregation prone stretches that are usually hidden in its hydrophobic core (Fig. 5C). In SAM domain mutants these stretches get exposed leading to aggregation of p63 and possible co-aggregation with other factors which is the underlying molecular mechanism of the AEC syndrome. These results further explain why frameshift mutations in the C-terminus of p63 also can cause the AEC syndrome. In all these cases, the newly synthesized sequences contain aggregation prone peptides [143].

### Transactivation inhibitory (TI) domain

The α-C-terminus of p63 contains a functional domain (amino acids 597–614) that is important for regulating the transcriptional activity of TAp63α [145]. The peptide itself is intrinsically disordered when isolated but forms a β-strand within the inactive dimeric structure of TAp63α (see below). Within the ΔNp63α isoform the TI domain remains accessible and can interact with the histone deacetylases HDAC1 and HDAC2 which contribute to transcriptional repression [146, 147]. C-terminal to the TI domain follows a sequence of 28 amino acids that is structurally not necessary for forming the inactive state of TAp63α [148] but contains a classical sumoylation site (IKEEGE) at the very C-terminus. Sumoylation of this site as well as other locations within the p63 sequence have been identified [149] and mutations that destroy the sumoylation site are linked to split-hand/foot malformation in human patients [150]. Mutational investigations in cell culture studies have suggested that sumoylation could reduce the cellular concentration of p63 [149, 151]. If this occurs via the SUMO-targeted ubiquitin ligase pathway [152–155] or other pathways remains to be investigated.

p73 contains a very similar sequence with a classical sumoylation site at its C-terminus (IKEEFT) as well. This site was shown to be the main modification site with SUMO in p73α by PIAS-1 [156] and sumoylation at the C-terminus results in faster proteasomal degradation [157]. Furthermore, sumoylation causes p73 to be preferentially found in detergent-insoluble fractions and thus might influence its subcellular localization, by targeting p73 for example to PML nuclear bodies as it is also observed for p53 [158] (however, the importance of sumoylation for subcellular targeting of p53 is debated [159]).

The TI domain of p63 also plays a role in patients with the AEC syndrome (see above in paragraph “Sterile-alpha-motif domain”). The TI domains of both p63 and p73 have a low intrinsic aggregation propensity [39]. Some of the AEC syndrome missense mutations that occur in the p63 TI domain enhance this aggregation propensity by removing “aggregation gatekeeping residues” that in the wild type protein inhibit aggregation [143]. The low aggregation tendency of the TI domains can also result in co-aggregation with the unfolded DBD of p53 carrying destabilizing cancer mutations [39].

## Isoforms

### ΔNp63α and ΔNp73α

ΔNp63α is an isoform that is expressed from a promoter situated upstream of exon 3′. Due to the shifted start site the N-terminal 69 residues of TAp63α are replaced with 14 unique amino acids [5]. The lack of the N-terminal TA domain (residues 8–25) reduces the transcriptional activity of ΔNp63α on promoters of classical p53 target genes such as *Ccgn1* (p21) or *Bax* [5]. Transcriptional activity has been measured on epithelial cell specific promoters such as *K14* [17, 160]. The function of ΔNp63α, however, seems to be not that of a classical transcription factor but that of an organizer of an epithelial cell specific chromatin landscape [161–166]. ChIP-seq experiments have revealed that this isoform binds to several thousand sites in the genome with a preference for enhancer and super enhancer sites [161, 167–174]. Interestingly, many of these sites differ between mouse and human keratinocytes and might be related to the observable phenotypic skin differences between both species [171]. To fulfill this function, a direct transcriptional activity is not necessary but interaction with the DNA as well as cooperation with other transcription factors [164]. This task requires high affinity DNA binding which is achieved by a constitutive open, tetrameric conformation in which the DBDs are flexibly tethered via the OD. NMR investigations have confirmed that the DBDs rotate freely and independently [148]. Similarly, ΔNp73α was shown to be an open tetramer competent to bind to DNA [175].

### TAp63α and TAp73α

**Fig. 6:**
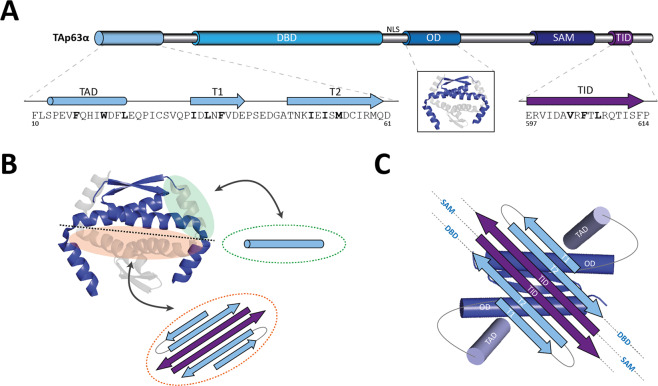
Model of the autoinhibitory complex of the inactive TAp63α dimer. **A** TAp63α is kept in an inactive dimeric conformation unable to bind DNA and transactivate target genes. Several (sub-)domains are involved in formation of the dimer: the transactivation (TA) domain, the β-strand T1, the β-strand T2, the oligomerization domain OD and the transcriptional inhibitory (TI) domain. The TA domain forms an α-helix, T1, T2 and the TI domain β-strands. The hydrophobic core motifs mediating the inter- and intramolecular interactions between these subdomains are highlighted in bold. **B** The current model of the inactive TAp63α dimer proposes the blockage of the tetramerization interface of the OD. The β-strands of T1, T2 and TI domain of two p63 molecules form a six-stranded antiparallel β-sheet that utilizes its hydrophobic side to interact with the also hydrophobic tetramerization interface of an OD dimer (orange). The helical TA domain simultaneously binds the interface of the OD which is normally bound by the second helix of the OD of the opposing dimer (green). Thereby both interfaces used for efficient dimerization of two OD dimers (blue and gray) to form a stable tetramer are blocked. **C** In the detailed model of the autoinhibitory complex, the six-stranded β-sheet sits on top of the tetramerization interface of the dimeric OD and both TA domains reach around the OD blocking the binding site of the second helix of the OD.

TAp63α is highly expressed in mammalian oocytes that are arrested in prophase of meiosis I [24, 32]. In mice its expression can be detected from day E18.5 on when oocytes have completed the repair of DNA double strand breaks (DSBs) induced by the enzyme Spo11 [176, 177]. These DNA DSBs get repaired by the process of homologous recombination [178] with some of the repair events resulting in cross-overs. These cross-overs are essential for a correct pairing of homologous chromosomes and thus reliable chromosome separation during meiosis. At the end of this process the expression of TAp63α increases and at day P5 virtually all oocytes have entered the dictyate arrest phase and show strong expression [24]. This expression level remains high until oocytes are recruited for ovulation. To ensure that during the long arrest phase—which can last in humans up to ~50 years—the pro-apoptotic transcription factor TAp63α does not induce cell death the transcriptional activity of this isoform is tightly controlled. This is achieved by adopting a closed and only dimeric conformation [179] (Fig. 6). While the structure of the inhibited state is not determined yet, extensive biochemical analysis has provided a detailed model of its conformation [148]. In this model the C-terminal TI domain plays a key role. Within the dimer, the two TI domains form an antiparallel β-sheet. This β-sheet gets extended by two strands (T1 and T2) from the sequence located between the TA and the DBD domains, thus creating a six-stranded antiparallel β-sheet [148]. The sequence containing the T1 and T2 β-strands correspond to the region of the TAD2 of p53 and p73 (see above). Interestingly, the interaction between the TI domain and one of the β-strands from the N-terminal sequence (T2) was confirmed by the Alphafold2 calculations, which however, are based on a monomer and can therefore only partially recapitulate all interactions necessary to stabilize the closed dimeric state [180]. The six-stranded antiparallel β-sheet has two completely different faces: one is very hydrophobic, the other one highly charged. According to the current model [148], the hydrophobic face interacts with the tetramerization interface of the OD, which is created by removing one of the dimers and is also hydrophobic (Fig. 6). Blocking this tetramerization interface prevents the interaction of two dimers via their helices. The position of the second helix that in the structure of the tetrameric OD interacts with elements of the other dimer (reaching across the tetramerization interface, see above and Fig. 4) [121–123] is currently not known and potentially it is even unfolded. The N-terminal α-helical TA domain is also important for stabilizing this closed conformation: it probably also binds to the OD, potentially occupying the position of the second helix of the OD. Mutating key hydrophobic residues (F16, W20, L23, these residues correspond to the amino acids that in p53 bind to Mdm2 [56]) within the TA domain destabilizes the conformation and results in the formation of an open tetrameric state [179]. As these amino acids are also key residues that interact for example with the Taz2 domain of p300 [61] and with other transcriptional activators, burying them in the closed state blocks interaction with the transcriptional machinery. In addition, the DNA binding affinity is strongly reduced [179]. By combining both effects—blocking interaction with the transcriptional machinery and inhibiting DNA binding—a strong total inhibition is achieved which explains why oocytes can survive for long times despite the high content of TAp63α.

**Fig. 7:**
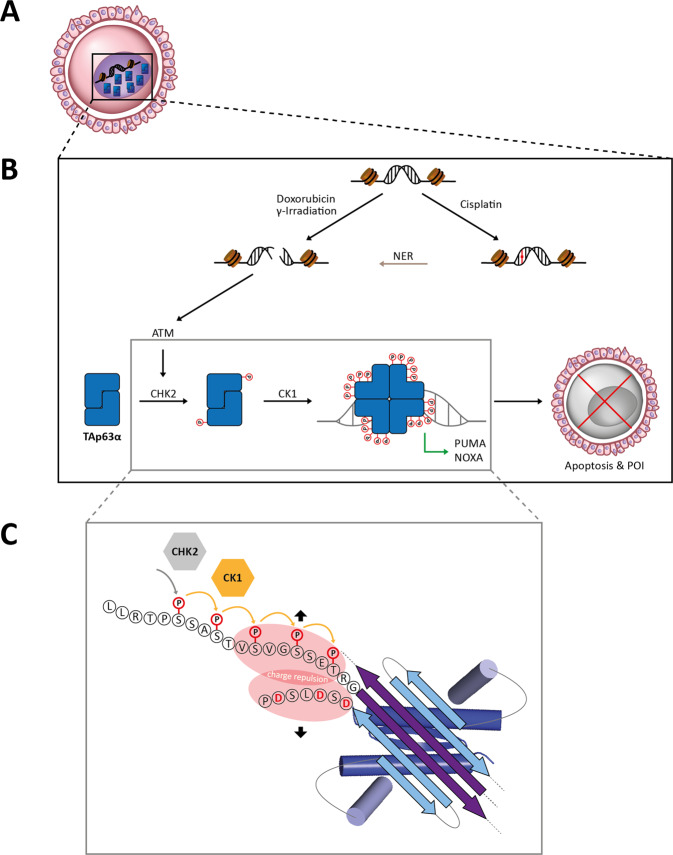
Model of the phosphorylation dependent activation of TAp63α. **A** The dimeric and inactive TAp63α is highly expressed in oocytes during dictyate arrest. **B** Anti-cancer therapy with chemotherapeutic agents or γ-irradiation causes DNA damage. Doxorubicin or γ-irradiation directly induces DSBs. Cisplatin, however, creates covalent inter- and intra-strand DNA adducts, which are then turned into DSBs by the nucleotide excision repair (NER) pathway. The kinase ATM is recruited and activated by DSBs and activates its downstream kinase Chk2 by phosphorylation. Chk2 in turn phosphorylates TAp63α at a single residue (S582) creating a consensus sequence for the constitutively active kinase CK1. CK1 then consecutively phosphorylates S585, S588, S591 and T594 as with each step it creates a new consensus sequence to phosphorylate the next residue at position i + 3. The accumulated negative charge of the phosphate groups triggers the formation of an active tetramer inducing the transcription of the pro-apoptotic Bcl-2 family members PUMA and NOXA [181]. This leads to apoptosis of the primordial follicles and ultimately to premature ovarian insufficiency (POI) upon cancer therapy [188, 189, 230–232]. **C** The priming kinase Chk2 and the executioner kinase CK1 phosphorylate serine and threonine residues in a consecutive manner. In the autoinhibitory complex the phosphorylated sequence is adjacent to the TI domain and thereby in proximity to the negatively charged residues following the β-strand T2 (D61, D63 and D66). The charge repulsion with these residues breaks open the autoinhibitory complex leading to irreversible tetramerization.

Activation of this closed dimeric state to the open tetrameric state that can bind to DNA and initiate a pro-apoptotic program [181] requires phosphorylation (Fig. 7). The first kinase that was identified to phosphorylate TAp63α directly was checkpoint kinase 2 (Chk2) [182]. Chk2 phosphorylates S582 which is located in a loop connecting the SAM and TI domains. This phosphorylation itself does not influence the conformation, TAp63α remains in the closed dimeric state [183]. However, pS582 recruits another kinase, Casein kinase 1 (CK1) that adds four more phosphate groups sequentially (Fig. 7) [183]. CK1 usually requires pre-phosphorylated sites and adds phosphate groups at the i + 3 position relative to the initial phosphate [184–186]. In p63, this results in phosphorylated sites at S585, S588, S591 and T594. This accumulation of negative charge just N-terminal to the TI domain leads to electrostatic repulsion with a group of three aspartic acid residues located next to the T2 β-strand. Activation to the open tetrameric state is irreversible, removal of all phosphate groups in the tetrameric state does not reestablish a closed dimeric state [179]. Likewise, experiments with urea have shown that moderate amounts of the denaturant result in the formation of the tetrameric state (without unfolding of the DBD, OD or SAM domains) but subsequent removal of urea does not lead to closed dimers [148]. These experiments have shown that the closed dimeric state of TAp63α constitutes a kinetically trapped high energy state and that the activation process follows a spring-loaded activation mechanism [148].

This irreversibility of the activation paired with the importance of the process (excessive oocyte death does not threaten the individual but the next generations) requires tight regulation. Surprisingly, only Chk2 is activated (by ATM) while CK1 is thought to be a constitutively active kinase [186]. This would make the decision about life and death in oocytes be dependent only on the activation state of Chk2. Detailed kinetic studies have, however, shown that the third phosphorylation event by CK1 (phosphorylation of S591) is significantly slower than phosphorylation of the first two sites (S585, S588) [187]. At the same time, this third phosphorylation event is required for tetramerization, while the fourth one (T594) is dispensable [183]. Activated, tetrameric TAp63α gets degraded fast and thus the kinetics of activation sets the threshold of DNA damage required for eliminating the compromised oocyte.

Most likely, the original role of TAp63α was to eliminate all oocytes that had not repaired the Spo11 induced DNA DSBs at the beginning of the dictyate arrest phase. However, TAp63α remains expressed in oocytes throughout the entire dictyate arrest phase and DSBs caused by other processes during that period result in activation of TAp63α. This is in particular a problem for patients treated for cancer or autoimmune diseases with chemotherapy or irradiation [24, 183, 188–193]. Activation of TAp63α in oocytes results in the loss of the pool of primary oocytes and thus leads to infertility and premature induction of menopause (Fig. 7). The dramatic effect of activated TAp63α for female fertility has also been demonstrated by the identification of mutations that create constitutively tetrameric (and therefore activated) TAp63α forms in human patients suffering from premature ovarian insufficiency [163, 194–197].

The high sequence identity between the p63 and p73 TI domains suggested that TAp73α might also adopt a closed and only dimeric conformation. Analysis of the oligomeric state, however, revealed that all p73 isoforms are constitutively tetrameric [175]. Domain swap experiments between p63 and p73 further demonstrated that replacing the p63 TI domain in TAp63α with the p73 TI domain leads to a closed dimeric conformation of the chimeric protein, proving that the p73 TI domain is in principle capable of supporting a closed state [61]. Further investigations have shown that the decisive difference between p63 and p73 is the N-terminal region and in particular residues 2–69. In p73 this sequence contains the bipartite TA domain while in p63 this sequence forms the N-terminal TA domain and the β-strands T1 and T2, which are part of the inhibitory β-sheet. Replacing the N-terminal region of TAp73α with the corresponding p63 sequence indeed resulted in the formation of a closed dimeric state of p73 [61]. These results also demonstrate that during evolution an originally inhibitory element (the β-strands) in p63 became an activating element (the second TA domain) in p73. This second TA domain finally became the dominant one in p53, while the original TA domain adopted an important role in regulation of p53’s activity via binding to Mdm2 [56].

### TA*p63α and GTAp63α

TAp63α is not the longest p63 isoform. Using a start site in exon 1, TA*p63α has an N-terminal extension of 39 amino acids (the transcriptional start site for TAp63α is in exon 2 [5]). Biophysical investigations have demonstrated that this extension stabilizes the closed dimeric state relative to TAp63α and at the same time provides a higher transcriptional activity in the open tetrameric state [198]. Thus, it seems to enhance the difference between the off- and the on-state of p63. Structurally, two helices are predicted for the extra 39 amino acids and are also predicted to form a TA domain. The exact function of this isoform and where it is expressed is currently not known. It can be detected on the mRNA level in different cells and at the protein level for example in the SUM159 cancer cell line [198]. Interestingly, a recent publication has shown that mutation of the start codon of TA*p63α causes a syndrome in human patients characterized by cleft tongue and muscular hypotonia, suggesting that the TA* isoforms might play a role during development [199].

A similar isoform is GTAp63α. It differs from TA*p63α by replacing the first 21 amino acids with 19 unique residues [198]. Both isoforms, however, share the 18 amino acids directly N-terminal to the start of TAp63α. The GTAp63α isoform was identified in male germ cells of humans and great apes and is created by incorporation of the 5′ LTR sequence of the human endogenous retrovirus 9 (ERV9) into the genome [200] which created an upstream exon, named U1. It might play a similar role in genetic quality control in male germ cells as TAp63α in female oocytes. In mice, however, p63 does not seem to be important in male germ cell quality control as activating mutations that create constitutively tetrameric and active p63 forms render female mice infertile but do not affect fertility of male mice [194, 201].

For p73 similar N-terminal extensions have so far not been reported.

### TAp63β and TAp73β

The β-isoforms of p63 and p73 are created by alternative splicing at the 3′ end of the p63 mRNA which removes at the protein level the SAM domain except for the three N-terminal amino acids of the first α-helix. This splicing also removes the inhibitory TI domain and thus makes all β-isoforms constitutively tetrameric. While no physiological functions for the p63β proteins are known (although mRNA transcripts are found in several tissues [30]), the TAp73β isoform is expressed in several tissues along with TAp73α. The specific function of the p73 α-C-terminus was investigated with a mouse model in which the exon 13 was deleted, thus expressing only the β-isoforms [202]. This mouse model (*Trp73*^*Δ13/Δ13*^) suffers from severe hippocampal dysgenesis, reduced synaptic functionality and impaired learning and memory capabilities caused by the depletion of CR cells [202]. In contrast to these neuronal defects no effect was seen for the development of the airway ciliated epithelium, suggesting that the α-C-terminus—and in particular the SAM and TI domains—is dispensable for multiciliogenesis [203].

### TAp63γ and TAp73γ

The γ-C-terminus is created by alternatively splicing of exon 10 to the γ-specific exon 10′, thereby adding 38 γ-specific residues directly C-terminal to the OD [5]. For p63 this isoform was shown on the mRNA level to be the dominant isoform in skeletal muscle [30]. If the γ-isoforms are also expressed on the protein level, is, however, not known. Several publications have discussed roles of TAp63 in skeletal and cardiac muscle development and disease [21–23, 30] as well as preventing aging and maintaining adult skin stem cells [204], the regulation of Dicer [31] as well as induction of senescence and suppression of tumorigenesis [205, 206]. Unfortunately for most studies the exact C-terminal splice isoform was not identified (it could be either TAp63α, TAp63β, TAp63γ or a mixture of all). In these studies inactivation of TA-specific isoforms were reported to change the transcriptional profiles of the investigated tissues and cells. As TAp63α would most likely adopt an inactive dimeric conformation these changes of the transcriptional profile suggest that TAp63γ (or maybe TAp63β) is the dominant isoform in these processes. Changes in the transcriptional profile would be difficult to explain if the transcriptionally repressed TAp63α isoform would be the dominant one. How TAp63γ that is at least in cell culture constitutively tetrameric, transcriptionally very active and pro-apoptotic [5, 179] contributes to the described processes and the precise role of the short γ-C-terminus remains to be studied. For the p73γ isoforms no physiological function has been convincingly demonstrated yet. Likewise, a physiological function for all even shorter p63 and p73 isoforms still has to be identified (although in particular for p73 shorter isoforms are detected on the mRNA level in different tissues [29, 30]).

## Evolution of the p53 protein family

The identification of p53 homologs in short lived invertebrates has sparked the interest in the evolution of the p53 family [48, 207–212]. In these organisms, tumor suppression is due to the short life span that prevents the accumulation of mutations, the limited amount of renewable tissues and the early sexual maturation and reproduction not a relevant problem. Consequently, the p53 homolog in *C. elegans*, Cep-1 [213, 214], for example, is found in the germ cells of this worm, strongly suggesting that germ cell quality control is the origin for the development of the entire p53 family. As germ cells are potentially immortal—they are not only the source for the somatic cells of the next generation but also for the germ cells of all following generations—keeping the genetic information of oocytes (and potentially also sperm cells) under strict quality control became advantageous. Once the life span of the organism started to exceed the life span of individual cells and evolution “invented” renewable tissue, the occurrence of somatic tumors became an evolutionary pressure which resulted in the development of p73 and in particular of p53 as tumor suppressor genes.

**Fig. 8:**
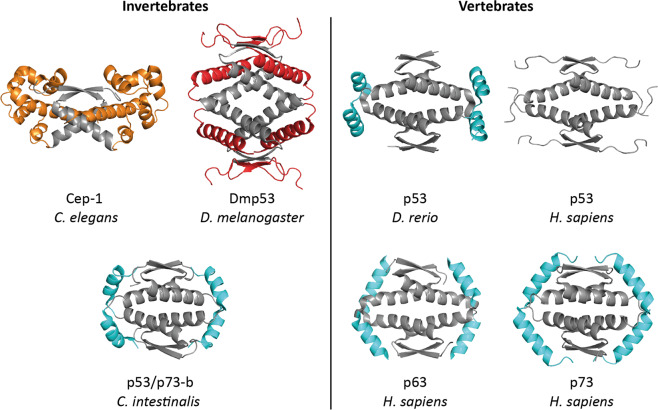
Structural comparison of the ODs of different invertebrate and vertebrate species. The structures of the p53 ODs of different invertebrate species are compared with the ODs of human p53 (PDB code: 1SAF), p63 (PDB code: 4A9Z) and p73 (PDB code: 2KBY) as well as with p53 from *Danio rerio* (zebrafish; PDB code: 4D1M). In the structure of Cep-1 from *Caenorhabditis elegans* (PDB code: 2RP5) the OD forms only a dimer that is tightly coupled to a SAM domain (yellow). In the structure of the OD from *Drosophila melanogaster* (PDB code: 2RP4) each secondary structure element known from human p53 is doubled. The extra β-sheets and α-helices per monomer are shown in red. The OD of p53/p73-b from the tunicate *Ciona intestinalis* (PDB code: 2MW4) is structurally similar to human p63 and p73 showing a second helix, despite the replacement of amino acids crucial for forming this second helix in vertebrate species. The structure of the p53 OD from *Danio rerio* contains a second helix that, however, is differently orientated compared to the second helices in human p63 and p73.

This hypothesis also means that the function of TAp63α in oocytes evolutionary precedes the function of ΔNp63α in epithelial stem cells. Structure determination of the folded domains of the *C. elegans* p53 homolog Cep-1 support the hypothesis of the germ cell quality control by p63 being the evolutionary origin. These investigations have shown that the DBD is again very similar to the DBDs of p53 and p63 with a similar DNA sequence preference [215]. The OD, however, forms only constitutive dimers that do not have the ability to create tetramers (Fig. 8) [216]. Instead, the OD is structurally coupled to a SAM domain that in mammalian family members exists in p63 (and p73) but not in p53. The Cep-1 SAM and OD domains are linked by a helix with extensive interactions with both parts and removal of the SAM domain destabilizes the OD. The inability to form tetramers is based on the replacement of important hydrophobic amino acids in the tetramerization interface of the p53 OD (M340, A347, and L348) with charges amino acids (K544, R551, and E552). Mutation of these charged amino acids to hydrophobic residues induces tetramerization [216].

Other invertebrate p53 homologs have evolved additional variations of the OD. In the OD of Dmp53 [217–219], the homolog of *Drosophila melanogaster*, each secondary structure element of the p53 OD is doubled: each monomer not only comprises an additional C-terminal helix as is the case in the ODs of p63 and p73, it contains also an additional N-terminal β-strand (Fig. 8). In the case of the Dmp53 OD each of these additional secondary structure elements is necessary for the stability of the entire domain. In contrast to p63 and p73, the second helix does not reach across the tetramerization interface but is located antiparallel to the first helix of each monomer.

In germ cells of Drosophila, different Dmp53 isoforms that differ by the length of the N-terminus and that are created by the use of alternative promoters and RNA splicing have been found [220, 221]. Only the shorter isoform (called Dmp53A) activated apoptosis in response to ionizing radiation [222], suggesting that autoinhibitory and regulatory elements likely exist in this insect family member as well.

While the existence of the N-terminal β-strand in the Dmp53 OD and the SAM domain at the C-terminus of the Cep-1 OD are so far unique features of these proteins, a second helix within the OD has been found in many other members of the p53 family [210]. Vertebrates have in general all three family members. Interestingly, structure determination of the OD of p53 from zebrafish has shown that it also contains a second helix, that is, however, tilted by 50° relative to the second helix in p63/p73 (Fig. 8) [210]. Further sequence analysis suggests that some bony fish retain the second helix in their OD while it was lost in other fish families (*Acanthomorpha*, spiny-rayed fishes) [210]. As the second helix in p53 is also missing for example in mammals, it seems that the second stabilizing helix was lost independently several times during evolution [210] and replaced with a disordered region that in human p53 is used as an integrator hub for countless posttranslational modifications and that is probably linked to acquiring additional functionality in somatic cells [223–226]. Stability of the core p53 OD was achieved by tighter packing and the establishment of the crucial Arg337-Glu352 salt bridge that stabilizes the primary dimer and thus also supports the formation of stable tetramers even without the additional helix [210].

Gene duplication and evolutionary loss of the second helix occurred also in invertebrates. Structural information on the OD is available for the two p53 family members found in *Ciona intestinalis* [209, 227], an organism that belongs to the tunicates and that represents the closest living relatives of vertebrates [228]. The C.int. p53/p73-a named protein does not contain a second helix while the C.int. p53/p73-b named protein contains the second helix [229]. This second helix, however, lacks the typical signature amino acids that are known to form the second helices of vertebrate p53 [210]: the N-terminal Pro residue that caps the helix is shifted by one position in the Ciona protein and a Tyr-Arg di-peptide toward the end of the helix is replaced with a Cys-Cys sequence [229]. Interestingly, the sequence that directly C-terminally follows the OD of C.int. p53/p73-a and that is disordered at 25 °C becomes more rigid at 10 °C indicating that a second helix in this region might form at lower temperature [229]. As helix formation could in principle also be initiated by posttranslational modifications, this observation suggests that in some species a stabilizing second helix might get induced and stabilization of tetramers might be posttranslationally regulated.
